# Gold Nanorod-Assisted Photothermal Therapy Decreases Bleeding during Breast Cancer Surgery in Dogs and Cats

**DOI:** 10.3390/cancers11060851

**Published:** 2019-06-19

**Authors:** Moustafa R. K. Ali, Haithem A. M. Farghali, Yue Wu, Ivan El-Sayed, Ahmed H. Osman, Salah A. Selim, Mostafa A. El-Sayed

**Affiliations:** 1 Laser Dynamics Laboratory, School of Chemistry and Biochemistry, Georgia Institute of Technology, Atlanta, GA 30332-0400, USA; janewuyue@gmail.com; 2 Department of Veterinary Medicine, Cairo University, Giza 12211, Egypt; haithemfarghali@gmail.com (H.A.M.F.); AHOsman2007@hotmail.com (A.H.O.); dr.salahselim@hotmail.com (S.A.S.); 3 Department of Otolaryngology, University of California, San Francisco, CA 94115, USA; Ivan.El-Sayed@ucsf.edu

**Keywords:** plasmonic photothermal therapy, gold nanorods, surgery, bleeding, dogs, cats, breast cancer

## Abstract

For localized tumors, gold nanorod (AuNR)-assisted plasmonic photothermal therapy (PPTT) is a potentially effective alternative to traditional surgery, in which AuNRs absorb near-infrared light and convert it to heat in order to kill cancer cells. However, for large tumors (volume ≥ 20 cm^3^), an uneven distribution of AuNRs might cause inhomogeneity of the heat distribution inside the tumor. Surgery is frequently recommended for removing large tumors, but it is associated with a high risk of cancer recurrence and metastasis. Here, we applied PPTT before surgery, which showed improved treatment for large tumors. We divided the animals (eight cats/dogs) into two groups: Group I (control), where three cases were solely treated with surgery, laser, or AuNRs alone, resulting in recurrence and metastasis; and Group II, where animals were treated with PPTT before surgery. In Group II, four out of the five cases had tumor regression without any recurrence or metastasis. Interestingly, we observed that applying PPTT before surgery displayed reduced bleeding during tumor removal, supported by histopathology that showed altered blood vessels. In conclusion, our study showed that applying AuNR-assisted PPTT (AuNRs-PPTT) before surgery could significantly affect blood vessels inside the tumor, leading to a decreased amount of bleeding during surgery, which can potentially decrease the risk of metastasis and blood loss during surgery.

## 1. Introduction

Gold nanorods (AuNRs)-based plasmonic photothermal therapy (PPTT) is a cancer therapy in which AuNRs are injected into the tumor before exposure to near-infrared (NIR) light [1,2]. The NIR light capable of deeply penetrating the tissue is transiently applied to the tumor, producing localized heat that could lead to tumor necrosis and apoptosis [3]. PPTT modulation to induce cell apoptosis might be a more favorable option than triggering necrosis, as during necrosis, the broken plasma membrane leads to the leaking of cytoplasmic components and inflammation, which could further induce cancer growth and metastasis [4,5,6,7]. The high efficiency of PPTT in getting rid of cancer cells by inducing apoptosis has been demonstrated both in vitro and in vivo [3,8]. The reason for using canines and felines is because of their molecular and biological similarity to human mammary tumors, which makes these animals a model system [9,10]. The surgery is usually the first line of treatment in the animals. In many animal cases, the tumors often metastasize. We have shown the efficacy of this treatment in tumor-bearing mice [8,11] and have recently demonstrated the treatment of mammary carcinoma in dogs and cats, where the malignant mammary tumors of ≤20 cm^3^ in volume could be treated by intratumoral inoculation of AuNRs, followed by PPTT [5]. During these studies, we observed that animals with induced or spontaneous tumors have noteworthy regression without any recurrence nor metastasis. Our recent studies have also shown the ability of gold nanorod-assisted plasmonic photothermal therapy (AuNRs-PPTT) in vitro to inhibit cancer cell migration [12,13]. In addition, the toxicity of this treatment has been examined in mice, dogs, and cats, which indicates that there are no toxic effects on the animals for long periods of time [5,8]. Therefore, PPTT is believed to be a favorable alternative for treating solid tumors with relatively small sizes and preventing metastasis [14]. 

For large tumors (volume ≥ 20 cm^3^), PPTT could be hard to achieve because of an uneven distribution of injected AuNRs that causes an inhomogeneity of the heat in the tumor. In these cases, surgery is usually recommended to remove primary solid tumors. However, the surgical resection of primary breast cancer tumors commonly has a risk for metastatic recurrence [15]. It has been recognized that the tumors contain large amounts of blood vessels that provide nutrients that support tumor growth [16]. Blood vessels are also critical for metastasis, as extensive and highly permeable blood vessels provide ways for cancer cells to exit primary tumor sites and enter into the bloodstream. Surgery usually disrupts the blood and lymphatic vessels, thus releasing cancer cells into the vasculature and promoting metastasis [17]. Therefore, the development of novel therapies that aid in the surgical process to prevent blood loss, tumor recurrences, and metastasis are of great importance. 

In this study, we are focusing on introducing a new treatment regimen featuring the combination of PPTT and surgery in dogs and cats with large tumors, as well as examining the efficacy of this new regimen. Interestingly, we observed that applying AuNRs-PPTT before surgery could significantly decrease bleeding, which could potentially avoid the risk of metastasis caused by surgery. 

## 2. Results

### 2.1. Preparation and Characterization of the AuNRs 

**Figure 1:**
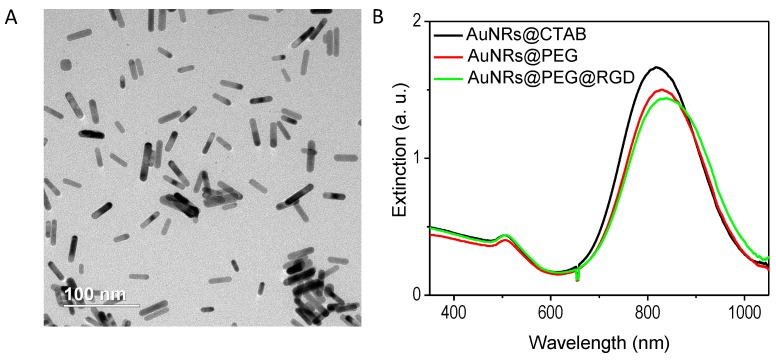
Characterization of gold nanorods (length 27 ± 5 nm, width 6 ± 1 nm). (**A**) Transmission electron microscope (TEM) image with 100 nm scale bar. (**B**) UV–VIS absorbance spectra showing the surface plasmon resonance peaks of gold nanorods (AuNRs) after synthesis (AuNRs@CTAB (cetyltrimethylammonium bromide)), then after conjugation with polyethylene glycol (PEG) (AuNRs@PEG), then after conjugation with (Arg−Gly−Asp) RGD (AuNRs@PEG@RGD).

AuNRs with an average size of 27 (±5) × 6 (±1) nm (length × width) were used in this study, as they showed enhanced efficacy of PPTT [18]. AuNRs were synthesized according to our reported method [19], and these AuNRs are shown in the transmission electron microscope (TEM) image (Figure 1A). AuNRs have a surface plasmon resonance wavelength of around 800 nm (Figure 1B). After synthesis, AuNRs were successfully coated with Methoxy polyethylene glycol thiol (mPEG-SH) and Arg−Gly−Asp (RGD) peptides, as demonstrated by the red-shift of the surface plasmon peak (Figure 1B). The average number of ligands on each particle is quantified to be 1000 mPEG-SH and 10,000 RGD. RGD is known to bind to integrin, which is over-expressed on the surface of breast cancer cells and associated with breast cancer progression and metastasis [20]. RGD binding could enhance the receptor-mediated endocytosis of the nanoparticles [21].

### 2.2. PPTT Decreases Bleeding during Surgery 

In our earlier studies, we optimized the PPTT conditions for treating dogs and cats, including the AuNR dosage and laser conditions (7.5 nM of AuNRs irradiated by NIR laser with 0.5 W/cm^2^ intensity for 2 min). The optimized conditions were conducted multiple times (2 weeks apart) until complete regression via apoptosis was shown, and this apoptosis proved to be better than necrosis [5]. Herein, we devised a new treatment regimen for treating animals with tumor volumes ≥20 cm^3^ by combining surgery with PPTT. 

**Figure 2:**
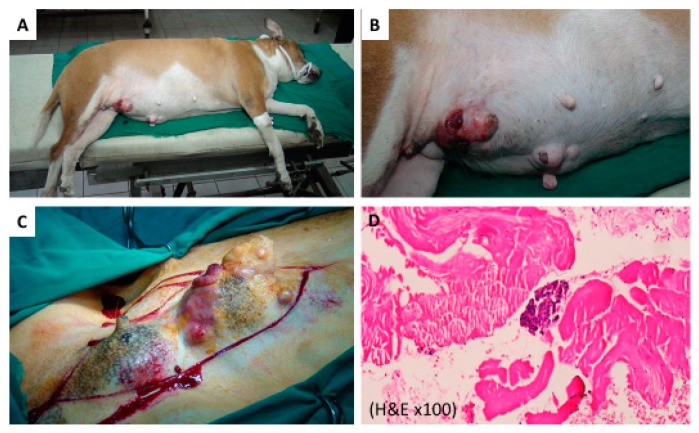
(**A**) Photographic image of control case 1 (Group 1, case 1) treated with surgery. (**B**) A magnified figure of (A) for the tumor area. (**C**) Photo of case during surgery with high amount of bleeding. (**D**) Photomicrograph of tumor tissue, showing ductal carcinoma in situ grade II (H&E ×100). Three images for each tumor were evaluated.

Before treatment, all animal tumors showed varied growth, as shown in Table 1 and Table 2. In the control group (I), three cases with 10 tumors were solely treated by a mastectomy, laser, or AuNR treatment alone. As shown in Figure 2A,B, photographic images of case 1 (GI-1) revealed three large tumors located at the cranioabdominal and inguinal lymph nodes. All three tumors connected to form one chain. Figure 2C is a picture of a tumor during surgery with obvious bleeding (over 100 g). Figure 2D is a histopathology of the tumor tissue showing ductal carcinoma in situ, grade II. 

**Figure 3:**
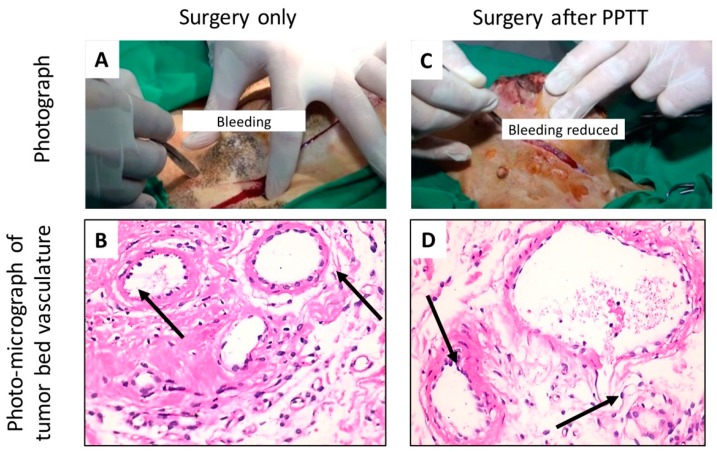
Left side (**A**,**B**) shows case 1 in Group I (surgery only). Right side (**C**,**D**) shows one case in Group II (treated with PPTT for three sessions (2 week intervals) before the surgery). (**A**,**C**) Photographic images indicating the decrease of bleeding after PPTT (**C**), compared with the control (**A**). (**B**,**D**) Photomicrograph of tumor bed vasculature showing (**B**) normal intact blood vessels with surgery only, and (**D**) swelling and sloughing of the endothelial lining and the destruction of the blood vessel wall (arrow) after PPTT (H&E ×400). Three images for each tumor were evaluated.

In Group II, five cases with eleven tumors were treated with three sessions of PPTT (in 2 week intervals) and were followed by surgery after the last PPTT session. Figure 3A,B shows case 1 (GI-1), where the animal was treated with surgery only, and Figure 3C,D shows one case with surgery after PPTT. Interestingly, we observed that when applying PPTT before surgery, reduced bleeding during the surgery was observed for all of the treated tumors in Group II when compared to Group I (Figure 3A,C and Videos S1 [surgery only] and S2 [surgery after PPTT]). 

The decrease of bleeding might be explained by the histopathology of the tumor bed vasculature (Figure 3B,D). With surgery only, the blood vessels were normal and intact (Figure 3B) while after PPTT, the tumor bed vasculature showed swelling and sloughing of the endothelial lining and destruction of the blood vessel walls Hematoxylin& Eosin (H&E ×400) (Figure 3D). 

**Figure 4:**
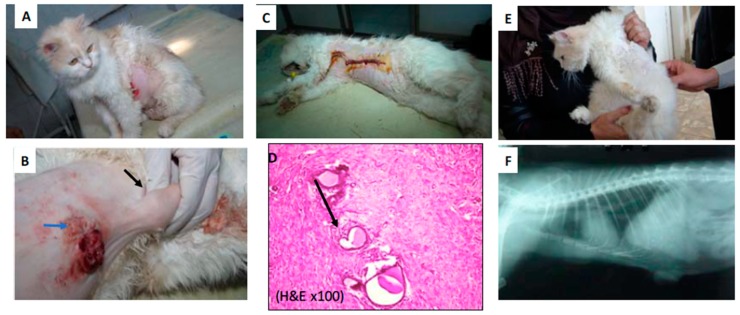
(**A**) A nine-year-old mixed breed cat suffered from mammary neoplasm (Group 2, case 1). (**B**) The sites of tumors at the left axillary lymph node (black arrow) and left cranial thoracic (blue arrow) (**C**) after surgical excision and (**D**) the subcutaneous layer showing well-developed granulation tissue at the site of suture (arrow) (H&E ×100). (**E**) The case after 12 months showed complete recovery from the surgery without any evidence of recurrence. (**F**) X-ray shows no metastasis in the chest. Three images for each tumor were evaluated.

In addition, the regimen of PPTT before surgery has shown to be effective for achieving complete tumor regression, as shown in Table 2. For example, in one case from Group II, a nine-year-old mixed breed cat suffered from mammary neoplasms as shown in Figure 4A. The site of two tumors located at her left axillary lymph node (black arrow) and left cranial thoracic (blue arrow) is demonstrated in Figure 4B. After PPTT, followed by surgical excision of the tumors (Figure 4C), tumor regression was achieved. The histopathology showed that after PPTT, well-developed granulation tissues were observed, indicating tumor recovery (Figure 4D). After 12 months, this case showed complete recovery from the surgery (Figure 4E) without evidence of recurrence or chest metastasis (Figure 4F). 

## 3. Discussion

Three cases among the five in Group II died a few months after PPTT and surgery treatment because of viral pneumonia, but evidence of tumor recurrence or metastasis was not observed. Earlier, we have shown in our study on mice that there was no toxicity after 15 months of AuNRs injection, whose bio-distribution mainly locates at the liver and spleen [8]. However, we do find that other types of nanoparticles, such as TiO_2_, iron, Cr^3+^-doped zinc gallate, and silver, could accumulate in the lung, and might cause toxicity [22,23,24,25], while AuNPs have better biocompatibility than these nanoparticles. To the best of our knowledge, there are not many systematic studies discussing the blood circulating (not airborne) AuNRs and their relation with pneumonia thus far. This can be a separate study in future.

In this report, we observed that PPTT affected the tumor blood vessels that, in turn, decreased the blood flow inside the tumor. The reason is still not fully understood. Previous reports have shown that AuNPs could affect blood vessels and tumor angiogenesis [26,27,28]. In addition, the temperature increase would cause the destruction of blood vessels. For instance, it has been reported that photothermal ablation of breast cancer in mice models using doxorubicin-loaded DNA-wrapped AuNRs could disturb the blood vessels [29]. Furthermore, it is reported that AuNPs with sizes around 30 nm could induce tumor endothelial leakiness [30]. In addition, the abnormal vascular nature of the tumor tissues allows them to uptake more AuNRs [31], which might explain why the AuNRs-PPTT is more effective on the tumor blood vessels. The importance of applying AuNRs-PPTT before surgery could also be very important in decreasing blood loss, especially for the patients who have injury-healing problems, including chronic diseases such as diabetes, and need tumor surgery.

## 4. Materials and Methods 

### 4.1. Synthesis and Surface Modification of AuNRs 

AuNRs were prepared according to the seedless method [19]. Briefly, 5 mL of 1 mM HAuCl_4_ (Sigma-Aldrich Co., St Louis, MO, USA) was mixed with 5 mL of 0.20 M cetyltrimethylammonium bromide (CTAB; Sigma-Aldrich Co.), followed by adding 250 μL of 4 mM AgNO_3_ (Sigma-Aldrich Co.) and adjusting the pH of the solution to be 1–1.15 by 37% HCl. Then, 70 μL of 78.8 mM ascorbic acid (Sigma-Aldrich Co.) was added to the solution until the solution became clear. A total of 15 μL of 0.01 M ice-cold NaBH_4_ (Sigma-Aldrich Co.) was injected into the growth solution immediately, and the solution was left unstirred for 6 hours. To remove the extra CTAB and prepare for surface modification, the AuNRs were centrifuged at 19,000 rcf for 1 hour, and the pellet was redispersed in deionized water and centrifuged at 14,000 rcf for 15 min. The AuNRs were rinsed with water, then conjugated with different surface ligands (polyethylene glycol (PEG) thiol and Arg−Gly−Asp (RGD) peptides). For surface modification, methoxy PEG thiol (m-PEG-Th, PEG; Laysan Bio, Arab, AL, USA) was added to AuNRs and stirred overnight to achieve a concentration of around 1000 PEG molecules per AuNR. For preparation of the AuNRs@RGD, the PEGylated nanoparticles (1 nM) were treated with RGD (1 mM) to achieve 10,000 ligands on each AuNR. Afterward, the solution was kept overnight to be shaken at normal temperature, and the extra ligands were removed by centrifugation. A UV–VIS spectrometer was used to confirm the conjugation. 

### 4.2. Characterization of AuNRs

A JEOL 100 CX transmission electron microscope (TEM) (JEOL Ltd., Tokyo, Japan) was used to measure the size and homogeneity of the samples. A Cary 500 UV–VIS spectrometer (Agilent Technologies, Santa Clara, CA, USA) was used for measuring the absorbance of the AuNRs. To characterize the surface conjugation with PEG, a ZetaSizer 3000 HAS (Malvern Instruments, Worcestershire, UK) was used for measuring the surface Zeta potentials. In addition, Ellman’s reagents (Sigma-Aldrich Co.), which react with free-Thiol groups (calorimetrically measured at 412 nm), were used to quantify the number of PEG molecules boun to the surface of the AuNRs. 

### 4.3. Animal Diagnosis and X-ray Examination

All animals were handled in accordance with Association for Assessment and Accreditation of Laboratory Animal Care and Office of Laboratory Animal Welfare guidelines under the direction of the Institutional Animal Care and Use Committee at Cairo University. The pet animals were admitted to the Department of Surgery Clinic of the Faculty of Veterinary Medicine at Cairo University. This research was approved by the Institutional Animal Care and Use Committee (CU-IACUC) Cairo University (code: CU II F 9 16).

All pets’ owners claimed that their animals did not receive any treatment before their arrival at the university. Written informed consent was provided by the owners of the pets for the treatments. Eight female animals were treated in this study, including two canines and six felines, with a total of 21 tumors with varied grades (I to III). The tumor dimensions were measured using calipers. Histopathology tests were used to diagnose the tumor types/grades. At the tumor site, the animal’s hair was shaved, and subsequently, radiographic recordings were taken with an X-ray machine (Fischer, Berlin, Germany). The radiographic setting factors were 58–70 kVp, 10 mAs, and a 90 cm focal spot film distance. The radiographic exposures were conducted dorsoventrally and right laterally. Blood loss was quantified by measuring the blood volume and weighing surgical sponges used for blood collection before and after the surgery. 

General anesthesia was applied for animals during the surgical mastectomy and postsurgical application of PPTT. Under general injectable anesthesia, each animal was pre-medicated with atropine sulphate (1%, 0.05–0.1 mg/kg b. wt.; Adwia Co. S.A.E., Cairo, Egypt) and xylazine (Xyla-Ject 2%, 1 mg/kg b. wt.; Adwia Co. S.A.E.), and then anesthesia was induced using ketamine HCl (Ketalar, 10–15 mg/kg b. wt.; Sigma-Aldrich Co.) and maintained by ketamine HCl [32,33]. 

### 4.4. Performing PPTT in Animals

Each animal was subjected to three sessions of PPTT treatment in 2 week intervals using an 808 nm diode laser with a power of 0.5 W/cm^2^ and a spot size of around 5.6 mm^2^. An effective dose of AuNR solution (7.5 nM AuNRs) for each 100 cm^3^ was used for 2 min, and the amount used was scaled up based on the volume of the tumor, and then injected directly into the tumor. Five minutes after injection, the entirety of the tumor was irradiated with the laser. The AuNR concentration was decreased by 50% for each subsequent treatment. The temperature increase of a tumor during the laser irradiation was measured by placing a 33-gauge hypodermic thermocouple (OMEGA Engineering, Inc., Stamford, CT, USA) needle directly inside the tumor (42–44 °C). 

### 4.5. Histopathology Evaluation of the Animal Tumors

The detailed pathologic evaluation of tumors was conducted by members of the pathology department of the Faculty of Veterinary Medicine at Cairo University. Histopathological analysis was performed on 5 μm sections from tumor tissue that were fixed in 10% buffered formalin. The samples were stained with H&E to assess pathology. 

## 5. Conclusions

Surgery is often used for tumor removal; however, it might trigger metastasis [34]. PPTT could be a better alternative to replace traditional chemotherapy and radiotherapy for localized tumors, especially for tumors with a volume ≤20 cm^3^ [5]. Therefore, for effective treatment of large tumors (volume ≥ 20 cm^3^), we applied PPTT before surgical resection to naturally occurring tumors in the mammary glands of dogs and cats. Five cases were treated with this regimen and showed complete remission without any recurrence after therapy. Three cases died in the few months following treatment, but in two cases from three, there was no evidence of any tumors upon examination (the animals died because of other reasons, such as pneumonia). Histopathology results showed a decrease in cancer grades compared to before (variant grades from 1 to 4) and after 2 weeks of treatment via PPTT and surgery (grade 0). X-ray diffraction revealed an absence of metastasis 1–2 years after treatment. In conclusion, our study demonstrates the feasibility of applying PPTT before surgery to large tumors in dogs and cats. Applying AuNRs-PPTT before surgery in treating large tumors could significantly affect blood vessels inside the tumor and potentially avoid the risk of bleeding during surgery. PPTT could be incorporated before the surgery to decrease the bleeding and potentially avoid the risk of bleeding during surgery that could lead to excessive blood loss and metastasis. 

## 6. Patents

This work has been filed in the US-Patent Publication of US20190008964A1.

## Figures and Tables

**Table 1:** Animal groups, tumor clinical features, and therapeutic approaches.

No. #	Species and Age	Site of Tumors	Size of Tumors (cm)	Grade	Therapy
GI-1	Dog—Mixed Boxer, 14 years	1-R caudoabdominal ulcerated	(8 × 5)	II	Only mastectomy
		2-R cranioabdominal	(18 × 4)		
		3-R inguinal Lymph node All the three connected to form one chain			
GI-2	Dog—Griffon, 7 years	1-R Cranioinquinal	(4.1 × 2.9)	II	Laser only, followed by mastectomy
GI-3	Cat—15 years	1-R caudo abdominal	(4 × 4.5).	II	1 + 2 + 3 tumors form chain; Laser only, followed by mastectomy
		2-R inguinal LN	(3 × 2.1).		
		3-L cranioabdominal	(5 × 2)		
		4-R inguinal	(1 × 2)	I	4 + 5 + 6 tumors treated by AuNRs only, followed by mastectomy
		5-L inguinal	(1 × 1)		
		6-L caudothoracic	(1 × 1)		
GII-1	Cat—9 years	L caudothoracic	(4.5 × 4)	III	Three sessions of plasmonic photothermal therapy (PPTT), followed by surgery
		R caudothoracic	(4 × 4)		
GII-2	Dog—Griffon, 10 years	R Caudoquinal	(3.1 × 3.6)	III	Three session of PPTT, followed by surgery
GII-3	Dog—Griffon, 11 years	1-L inguinal	(5 × 5)	II	Three session of PPTT, followed by surgery
		2-L inguinal	(2 × 1.5)		PPTT only
		3 small tumor	(1.5 × 1)		PPTT only
GII-4	Dog—Griffon, 5 years	1-R caudo thoracic	(3 × 2.5) (8 × 6.5)	II	Three sessions of PPTT, followed by surgery
		2-R cranio abdominal both tumors form chain			
GII-5	Dog—Griffon, 8 years	1-L inguinal large calcified	(5 × 5) (2 × 1.5) (1.5 × 1)	III	Three session of PPTT, followed by surgery
		2-small caudal abdomenial			2 and 3 treated by PPTT only
		3-two attached small tumors			

The control group, GI, included three cases (two dogs and one cat with 10 tumors) treated with either surgery only, laser only, or AuNRs only. All cases developed metastasis and died after treatment. Group II (GII) included five cases (four dogs and one cat with 11 tumors) treated with PPTT (AuNRs and laser together) for three sessions, followed by surgery. At the time of death, there was no presence of disease; however, one case succumbed to metastatic disease.

**Table 2:** Survival, bleeding loss, and metastasis for both GI and GII.

No. #	EBL (g)	LR	DM (Time Mo)	OSS (mo)	Status
GI-1	>100	+1 month	+(CS, LN)	3	DOD
GI-2	66.5	-	+(CS, LN)	1	DOC
GI-3	47.5	-	+(LN)	1	DOC
GII-1	<1	-	-	24	ADF
GII-2	<1	-	-	48	ADF
GII-3	<1	-	-	3	DOC (pneumonia)
GII-4	<1	-	+(LN, CS)	1	DOD
GII-5	<1	-	-	6	DOC (pneumonia)

Group I (GI) were solely treated with surgery, laser, or AuNrs alone. Group II (GII) were treated with PPTT (AuNrs and laser together) before surgery. Estimate blood loss significant test *p*-value and statistical significance: the two-tailed *p*-value = 0.0010—by conventional criteria, this difference is considered to be statistically significant. ADF, alive disease free; DM, distant metastases; CS, clinical stage; DOC, dead other cause; DOD, dead of disease; EBL, estimated blood loss; LN, lymph node; LR, local recurrence.

## Supplementary Materials

The following are available online at https://zenodo.org/record/3251162#.XR3I5_YRU2x, Video S1 [surgery only] and Video S2 [surgery after PPTT]).
